# System Identification Algorithm Analysis of Acupuncture Effect on Mean Blood Flux of Contralateral Hegu Acupoint

**DOI:** 10.1155/2012/951928

**Published:** 2012-05-24

**Authors:** Guangjun Wang, Jianguo Han, Gerhard Litscher, Weibo Zhang

**Affiliations:** ^1^Department of Biomedical Engineering, Institute of Acupuncture and Moxibustion, China Academy of Chinese Medical Sciences, Beijing 100700, China; ^2^Institute of Information Science and Technology, Beijing University of Chemical Technology, Beijing 100029, China; ^3^Stronach Research Unit for Complementary and Integrative Laser Medicine, Research Unit of Biomedical Engineering in Anesthesia and Intensive Care Medicine, and TCM Research Center Graz, Medical University of Graz, Auenbruggerplatz 29, 8036 Graz, Austria

## Abstract

*Background*. Acupoints (belonging to 12 meridians) which have the same names are symmetrically distributed on the body. It has been proved that acupoints have certain biological specificities different from the normal parts of the body. However, there is little evidence that acupoints which have the same name and are located bilaterally and symmetrically have lateralized specificity. Thus, researching the lateralized specificity and the relationship between left-side and right-side acupuncture is of special importance. *Methodology and Principal Findings*. The mean blood flux (MBF) in both Hegu acupoints was measured by Moor full-field laser perfusion imager. With the method of system identification algorithm, the output distribution in different groups was acquired, based on different acupoint stimulation and standard signal input. It is demonstrated that after stimulation of the right Hegu acupoint by needle, the output value of MBF in contralateral Hegu acupoint was strongly amplified, while after acupuncturing the left Hegu acupoint, the output value of MBF in either side Hegu acupoint was amplified moderately. *Conclusions and Significance*. This paper indicates that the Hegu acupoint has lateralized specificity. After stimulating the ipsilateral Hegu acupoint, symmetry breaking will be produced in contrast to contralateral Hegu acupoint stimulation.

## 1. Introduction

Acupuncture has been widely used to reduce some symptoms or to treat diseases in clinical practice for at least 2000 years [1]. During the past 30 years, a large number of studies focused on the antinociception mechanism of acupuncture, which made it more acceptable to clinical practice and mechanism research. According to the principles of Unschuld [2], acupuncture effects might be related to the appropriate acupoints during the treatment. However, previous studies have indicated that electroacupuncture (EA) is involved in modifying a variety of brain functions and in promoting the release of endogenous opioid peptides, which might be responsible for its analgesic effects in the whole body [3, 4]. It means that acupuncture specificity contributes a little to its effect. What is more, many researchers firmly believe that placebo effect may be the best explanation for acupuncture [5, 6].

On the other hand, specificity of acupuncture points seems to be confirmed by the evidence from neuroimaging studies [7, 8]. It has been shown that acupuncture at different acupoints induced differential hemodynamic neural responses in some brain areas [9]. In contrast, there is little evidence to discern the differences of acupoints which have the same name and are located bilaterally and symmetrically. Goldman et al. reported significant analgesic effects of ipsilateral but not contralateral acupoints in a mouse model of inflammatory pain [10]. Somers and Clemente [11] obtained opposite results: transcutaneous electric nerve stimulation on the contra- but not ipsilateral side of neuropathic pain resulted in antinociceptive effects in rats. Furthermore, a study [12] indicated that although the anti-nociceptive effect of both contralateral and ipsilateral EA was definitely confirmed, lesions of the rostral anterior cingulated cortex completely abolished the anti-nociceptive effects of contra- but not ipsilateral EA. These studies intensively suggested that there might be a difference between ipsilateral acupuncture and contralateral acupuncture.

In our lab in 1997, Zhang WB measured the transcutaneous CO_2_ emission on left and right 24 source acupoints and calculated the correlations between the points. It showed a significantly higher correlative coefficient (0.814) between the left and right same name acupoints than the correlative coefficient between general acupoints (0.379) [13]. Our recent studies have also shown that thermostimulation could result in an increase of blood perfusion not only in the local area [14] but also in the same area on the contralateral side [15]. This phenomenon can be observed both in the upper limb [15] and lower limb [16]. However, the same stimulation has no effect on periumbilicus area [14], which indicated that there might be intrinsic and symmetrical correlation between contra- and ipsilateral parts. This view was supported by Kubo et al. [17]. Their work indicated that after acupuncture or thermostimulation in the ipsilateral side, the blood volume increased gradually in contralateral Achilles tendon, and the amount of increase in blood volume of the nontreated tendon (contralateral side) was significantly correlated to that of the treated tendon (ipsilateral side) during the last phase of recovery period. Recently we reported that when either side Hegu acupoint (LI4) was stimulated, there was an increase in mean blood flux at LI4 of the contralateral side. However, the intrinsic correlation between contra- and ipsilateral LI4 is not clear. The purpose of this study is to investigate the correlation of bilateral LI4 through system identification algorithm analysis.

## 2. Methods

### 2.1. Ethics Statement

This study was reviewed and approved by the Institutional Review Board at the Institute of Acupuncture and Moxibustion, China Academy of Chinese Medical Sciences. Each participant read and signed an informed consent form.

### 2.2. Subjects

**Figure 1:**
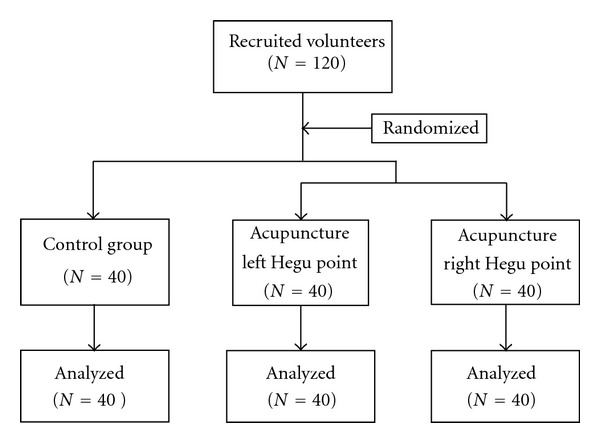
Flow diagram of participants in the study.

One hundred and twenty (120) healthy volunteers were recruited in this study (as shown in Figure 1; for demographic data see Table 1). All subjects were students from the China Academy of Chinese Medical Sciences and Beijing University of Traditional Chinese Medicine. All subjects had no history of diseases and had not taken any medicine in the past six months before the experiment. Each subject had an adequate understanding of the procedure and purpose of this study.

### 2.3. Procedures

#### 2.3.1. Protocol for Mean Blood Flux Measurement

**Figure 2:**
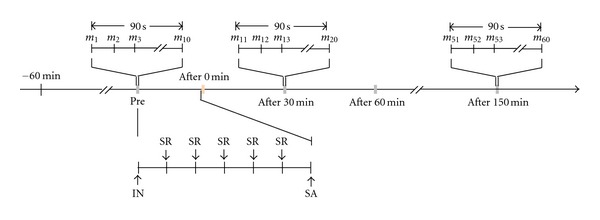
Procedure of acupuncture and mean blood flux measurement. Pre: pre acupuncture; Post, post acupuncture; IN: insert needle; SA: stop acupuncture; SR: slowly rotate the needle every five minutes; *m*_*i*_  (*i* = 1,2, 3,…, 60): mean blood flux of Hegu acupoint at a specific time point.

Before arrival to the laboratory, subjects were placed in a temperature-controlled room (24–26°C) as a resting state for 60 minutes. Measurements of skin blood perfusion were carried out using Moor full-field laser perfusion imager (moor FLPI, Moor Instruments Ltd, UK). Before recording, both hands were immobilized with a cylindrical object to ensure positioning. The measurement parameters were as follows: high resolution/250 frames; number of images = 10; exposure time = 8.3 ms; time interval = 10 s. Measurements were carried out every 30 minutes over a total of 180 minutes. During the experiment, the laboratory room was kept in dark light condition, and the protocol for measurement operation was abided strictly. The measurement process is illustrated in Figure 2.

#### 2.3.2. Acupuncture Protocol

For acupuncture, a small acupuncture needle, 0.25 × 25 mm (100112, Zhen Huan), was gently inserted in a depth of 15 mm in the LI4. The position of LI4 was confirmed according to the previous studies [18, 19]. The needle was slowly rotated every 5 min for a total of 30 min during an acupuncture session in order to maintain the soreness and numbness sensation of De-Qi [10]. The acupuncture procedure is illustrated in Figure 2. In the left acupuncture group (Left Acup.), just left LI4 was acupunctured whereas in the right acupuncture group (Right Acup.), right LI4 was stimulated. In the control group (No Acup.), all subjects maintained still, without any intervention.

#### 2.3.3. Image Analysis Protocol

**Figure 3:**
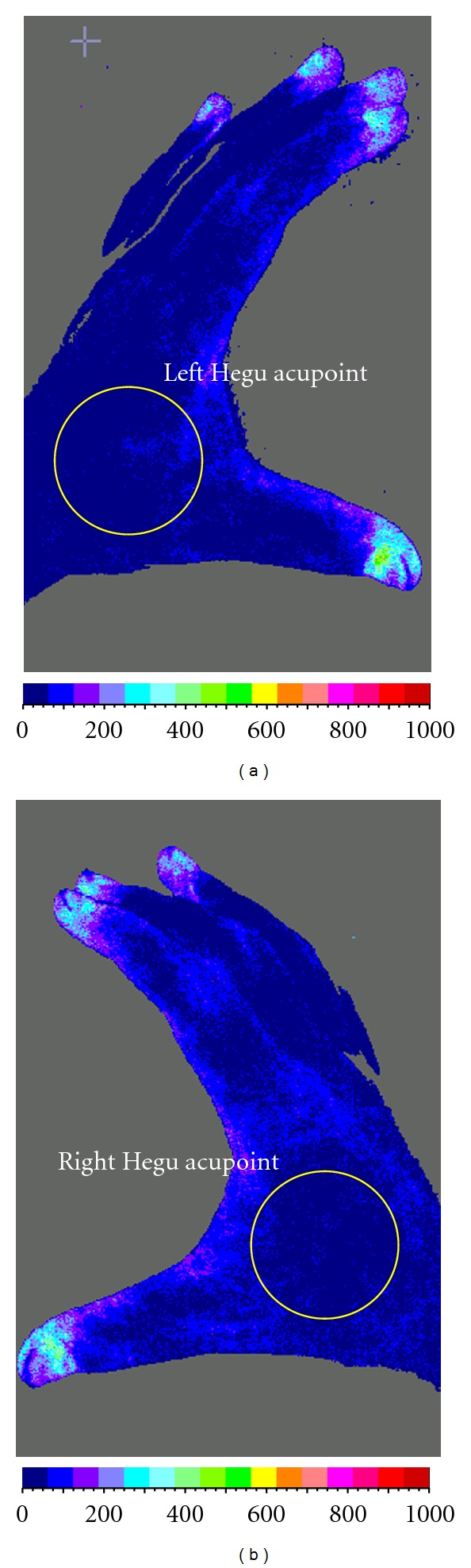
Confirmation of Hegu acupoint. (a) left hand, (b) right hand.

The Mean Blood Flux (MBF) of LI4 on both hands (left side abbreviated as L, right side abbreviated as R) was measured (Figure 3). In order to exclude the nonspecific effect of acupuncture practice, a total of 10 data from the post-0-minute phase were excluded from the final analysis (Figure 2). We symbolized left acupuncture group, right acupuncture group, and control group as A, B, and C, respectively. So for every person, there are 60 pairs of data acquired. For every group, there are 2400 pairs of data acquired. We denoted the mapping relationship of these data pairs, namely, mapping relationship from {*AL*⁡} to {AR}, from {BL} to {BR}, from {CL} to {CR} as {*AL*⁡} → {AR}, {BL} → {BR} and {CL} → {CR}, and summarized as $\left\{\begin{array}{c} \text{A}\\ \text{BL}\\ \text{C} \end{array}\right\}\to \left\{\begin{array}{c} \text{A}\\ \text{BR}\\ \text{C} \end{array}\right\}$.

#### 2.3.4. System Identification Algorithm

**Figure 4:**
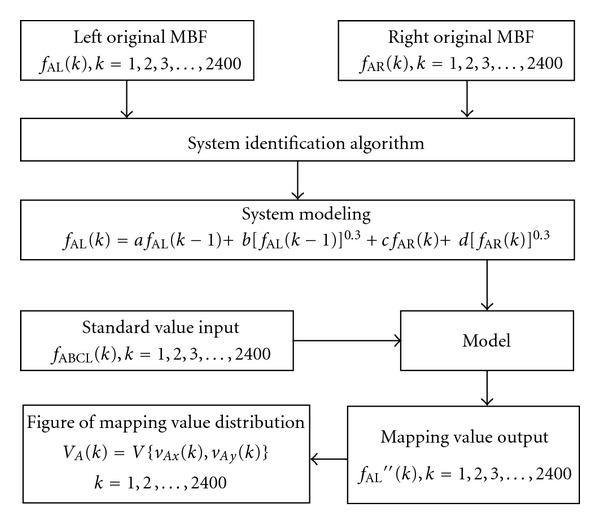
Flow diagram of system identification algorithm.

Under the condition of $\left\{\begin{array}{c} \text{A}\\ \text{BL}\\ \text{C} \end{array}\right\}\to \left\{\begin{array}{c} \text{A}\\ \text{BR}\\ \text{C} \end{array}\right\}$, totally 2400 × 3 pairs of data were acquired and symbolized as *f*_*AL*⁡_(*k*), *f*_BL_(*k*), *f*_CL_(*k*), *f*_AR_(*k*), *f*_BR_(*k*), *f*_CR_(*k*), *k* = 1,2,…, 2400, where A represents left acupuncture group; B represents right acupuncture group; C represents control group; L represents left Hegu acupoint; R represents right Hegu acupoint; *k* = 1,2,…, 2400. Then, we determined *f*_*AL*⁡_(*k*), *f*_BL_(*k*) and *f*_CL_(*k*), as input and *f*_AR_(*k*), *f*_BR_(*k*), and *f*_CR_(*k*) as output, respectively. System identification algorithm was performed in the Matlab software (Version: 6.5). The flow diagram of system identification algorithm is shown in Figure 4.

## 3. Results

### 3.1. Mapping Model

Three mathematical models were obtained as follows by executing system identification algorithm, which reflects the correlation of bilateral Hegu acupoints under different intervention conditions. 

Model 1:

(1) $$\begin{array}{cc} f_{\mathrm{AL}}\left(k\right) & =a_{1}f_{\mathrm{AL}}\left(k-1\right)+b_{1}{\left[f_{\mathrm{AL}}\left(k-1\right)\right]}^{0.3}\\ & \begin{array}{c} \;+c_{1}f_{\text{AR}}\left(k\right)+d_{1}{\left[f_{\text{AR}}\left(k\right)\right]}^{0.3}. \end{array} \end{array}$$

Model 2:

(2) $$\begin{array}{c} \begin{array}{cc} f_{\text{BL}}\left(k\right) & =a_{2}f_{\text{BL}}\left(k-1\right)+b_{2}{\left[f_{\text{BL}}\left(k-1\right)\right]}^{0.3}\\ & \;+c_{2}f_{\text{BR}}\left(k\right)+d_{2}{\left[f_{\text{BR}}\left(k\right)\right]}^{0.3}. \end{array} \end{array}$$

Model 3:

(3) $$\begin{array}{cc} f_{\text{CL}}\left(k\right) & =a_{3}f_{\text{CL}}\left(k-1\right)+b_{3}{\left[f_{\text{CL}}\left(k-1\right)\right]}^{0.3}\\ & \;+c_{3}f_{\text{CR}}\left(k\right)+d_{3}{\left[f_{\text{BR}}\left(k\right)\right]}^{0.3}. \end{array}$$

Using the values (measured on the left side) of *f*_*AL*⁡_(*k*), *f*_BL_(*k*), and *f*_CL_(*k*) as input variables of models (1), (2), and (3), the estimated value (mapping value) of the right Hegu acupoint can be obtained with help of the models, symbolized as *f*_AR_*(*k*), *f*_BR_*(*k*) and *f*_CR_*  (*k*), following the mapping relationship *f*_*AL*⁡_(*k*) → *f*_AR_*(*k*), *f*_BL_(*k*) → *f*_BR_*(*k*), and *f*_CL_(*k*) → *f*_CR_*  (*k*).

### 3.2. Error Evaluation and Signal-Noise Ratio

The errors between “true” value and their estimated values (symbolized as *d*_*i*_(*k*)) are defined as

(4) $$\begin{array}{c} d_{\text{A}}\left(k\right)=\;{f_{\text{AR}}}^{*}\left(k\right)-f_{\text{AR}}\left(k\right),\;k=1,2,\ldots ,2400,\\ d_{\text{B}}\left(k\right)={f_{\text{BR}}}^{*}\left(k\right)-f_{\text{BR}}\left(k\right),\;k=1,2,\ldots ,2400,\\ d_{\text{C}}\left(k\right)={f_{\text{CR}}}^{*}\left(k\right)-f_{\text{CR}}\left(k\right),\;k=1,2,\ldots ,2400. \end{array}$$

Then we defined the signal-noise ratio sn_*i*_  (*i* = *A*, *B*, *C*) as

(5) $$\begin{array}{c} {\text{sn}}_{\text{A}}=\frac{\left\{\sqrt{\sum_{k=1}^{2400}{\left[{f_{\text{AR}}}^{*}\left(k\right)\right]}^{2}}/\sqrt{\sum_{k=1}^{2400}{\left[d_{\text{A}}\left(k\right)\right]}^{2}}\right\}}{2400},\\ {\text{sn}}_{\text{B}}=\frac{\left\{\sqrt{\sum_{k=1}^{2400}{\left[{f_{\text{BR}}}^{*}\left(k\right)\right]}^{2}}/\sqrt{\sum_{k=1}^{2400}{\left[d_{\text{B}}\left(k\right)\right]}^{2}}\right\}}{2400},\\ {\text{sn}}_{\text{C}}=\frac{\left\{\sqrt{\sum_{k=1}^{2400}{\left[{f_{\text{CR}}}^{*}\left(k\right)\right]}^{2}}/\sqrt{\sum_{k=1}^{2400}{\left[d_{\text{C}}\left(k\right)\right]}^{2}}\right\}}{2400}. \end{array}$$

### 3.3. Standard Signal Input

Standard signal value series used as common model-input value series denoted as *f*_ABCL_(*k*) are mathematically calculated as

(6) $$\begin{array}{cc} & f_{\text{ABCL}}\left(k\right)\\ & =10\;\times \{\sin\left(\frac{k}{100}\right)+\sin\left(\frac{k}{80}\right)+\sin\left(\frac{k}{60}\right)\\ & \;\;\;\;\;+\sin\left(\frac{k}{40}\right)+\cos\left(\frac{k}{90}\right)+\cos\left(\frac{k}{70}\right)\text{+}\\ & \;\;\;\;\;+\cos\left(\frac{k}{50}\right)+\cos\left(\frac{k}{30}\right)\}\;k=1,2,\ldots ,2400. \end{array}$$

The standard signal input is to maintain the complexity and stability. If *f*_*AL*⁡_(*k*), *f*_BL_(*k*), and *f*_CL_(*k*) were all replaced by *f*_ABCL_(*k*), the output *f*_*AL*⁡_′′(*k*), *f*_BL_′′(*k*), and *f*_CL_′′(*k*) will be produced with help of the models, instead of *f*_AR_*(*k*), *f*_BR_*(*k*), and *f*_CR_*(*k*), respectively. Thus the mapping relationship will change into *f*_ABCL_(*k*) → *f*_*AL*⁡_′′(*k*), *f*_ABCL_(*k*) → *f*_BL_′′(*k*), *f*_ABCL_(*k*) → *f*_CL_′′(*k*).

### 3.4. Determination of Characteristic Vectors

In order to set up model output characteristic vectors to describe the different mapping results of different interventions, we define one subvector of the model output characteristic vector as

(7) $$\begin{array}{c} v_{\mathrm{AL}y}\left(k\right)={f_{\mathrm{AL}}}^{\prime \prime }\left(k\right),\;k=1,2,3,\ldots ,2400,\\ v_{\text{BL}y}\left(k\right)={f_{\text{BL}}}^{\prime \prime }\left(k\right),\;k=1,2,3,\ldots ,2400,\\ v_{\text{CL}y}\left(k\right)={f_{\text{CL}}}^{\prime \prime }\left(k\right),\;k=1,2,3,\ldots ,2400. \end{array}$$

The other subvector of the model output characteristic vector was determined as

(8) $$\begin{array}{c} v_{\text{A}x}\left(k\right)={\text{sn}}_{\text{A}}+d_{\text{A}}\left(k\right),\;\;k=1,2,\ldots ,2400,\\ v_{\text{B}x}\left(k\right)={\text{sn}}_{\text{B}}+d_{\text{B}}\left(k\right),\;k=1,2,\ldots ,2400,\\ v_{\text{C}x}\left(k\right)={\text{sn}}_{\text{C}}+d_{\text{C}}\left(k\right),\;k=1,2,\ldots ,2400. \end{array}$$

Then the 2-dimensional diagram of mapping value distribution was produced. In the left acupuncture group, the distribution of output was determined as

(9) $$\begin{array}{c} V_{\text{A}}\left(k\right)=V\left\{v_{\text{A}x}\left(k\right),v_{\text{A}y}\left(k\right)\right\},\;k=1,2,\ldots ,2400. \end{array}$$

In the right acupuncture group, the distribution of output was determined as

(10) $$\begin{array}{c} V_{\text{B}}\left(k\right)=V\left\{v_{\text{B}x}\left(k\right),v_{\text{B}y}\left(k\right)\right\},\;k=1,2,\ldots ,2400. \end{array}$$

In the control group, the distribution of output was determined as

(11) $$\begin{array}{c} V_{\text{C}}\left(k\right)=V\left\{v_{\text{C}x}\left(k\right),v_{\text{C}y}\left(k\right)\right\},\;k=1,2,\ldots ,2400. \end{array}$$

**Figure 5:**
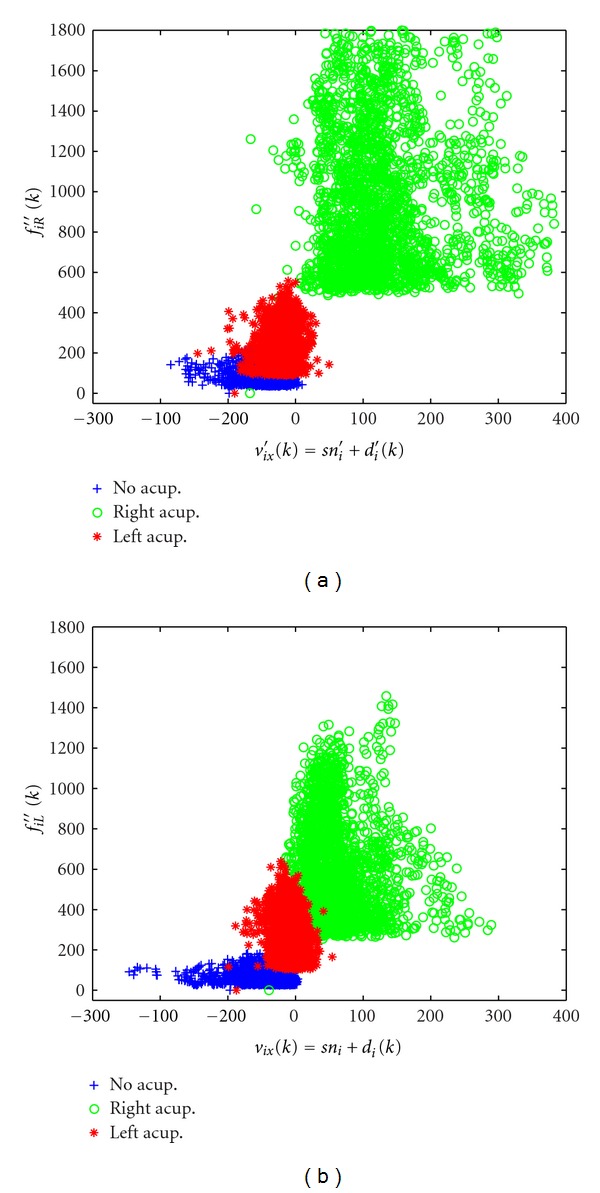
Mapping value distribution of standard input in different models.

The output distribution is shown in Figure 5(b). To exclude the possibility that these results were lateralized to one side, we defined the original *f*_AR_(*k*), *f*_BR_(*k*), *f*_CR_(*k*) as input, *f*_*AL*⁡_(*k*), *f*_BL_(*k*), *f*_CL_(*k*) as output, respectively, summarized as $\left\{\begin{array}{c} \text{A}\\ \text{BR}\\ \text{C} \end{array}\right\}\to \left\{\begin{array}{c} \text{A}\\ \text{BL}\\ \text{C} \end{array}\right\}$. Then the system identification algorithm was carried out with MATLAB software again, and the other 3 models were produced. When the same standard signals were input into the different models, the distribution of output was produced (Figure 5(a)). Mapping value distribution center and signal-noise ratio in different groups were shown in Table 2.

From Figure 5, we can find that stimulation of right LI4 has the strong amplification effect on blood perfusion in left LI4, and this strong amplification effect is independent of the original input and output selection in the system identification algorithm analysis. In contrast, acupuncture at left LI4 just produces moderate amplification effects on blood perfusion in right LI4, and this moderate amplification is independent of the original input and output selection too. There is no amplification effect produced in the control group. These results indicated that after acupuncture, the amount of amplification effect on blood perfusion in contralateral side was just related to which lateral acupoint was acupunctured. *i* = A, B, C, *k* = 1,2, 3,…, 2400. (A) under the condition of $\left\{\begin{array}{c} \text{A}\\ \text{BR}\\ \text{C} \end{array}\right\}\to \left\{\begin{array}{c} \text{A}\\ \text{BL}\\ \text{C} \end{array}\right\}$. (B) under the condition of $\left\{\begin{array}{c} \text{A}\\ \text{BR}\\ \text{C} \end{array}\right\}\to \left\{\begin{array}{c} \text{A}\\ \text{BL}\\ \text{C} \end{array}\right\}$. A, acupuncture left Hegu acupoint; B, acupuncture right Hegu acupoint; C, no acupuncture.

## 4. Discussion

“In physics, symmetry means uniformity or invariance” [20], in other words, “the existence of different viewpoints from which the system appears the same” [21]. In Traditional Chinese Medicine (TCM), the principle is to maintain the body balance. Under the guidance of TCM theory, clinical practice is always in the pursuit of balance and symmetry. For example, according to the Neijing theory, if someone has disease in the left body, the treatment point is usually selected in the right side, and vice versa. However, “increasing levels of broken symmetry in many-body systems correlates with increasing complexity and functional specialization” [20]. In acupuncture theory, the symmetry breaking means there are differences between two meridians or two acupoints which have the same name and are located bilaterally and symmetrically on the body.

Recently, a system review analyzed the contralateral and ipsilateral acupuncture effect on poststroke hemiplegic patients [22]. Although this system review and meta-analysis could not come to a definitive conclusion, it indicates the importance of distinction between contralateral and ipsilateral acupuncture. According to traditional acupuncture theory, if we stimulate LI4 on one side, the function of the large intestine meridian (LI) located on the other side might also be activated. As a result, the running of Qi and blood which flow in both LI meridians were changed. So the basis of contra- or ipsilateral acupuncture is the specificity of acupoints which have the same name.

But up to now, it is difficult to evaluate the activation of acupoints, and, as a result, it is also difficult to analyse the specificity of acupoints after meridians are stimulated. Recently, more and more attention has been focused on the relationship of acupuncture and circulation [23–25]. In TCM theory, one of the definitive causes of acupuncture effect is the special sensation in local acupoints after stimulation, which might be related to the blood perfusion changes in acupoints or meridians [18]. According to the previous study, the mean blood flux (MBF) was larger at the acupoints than in their surrounding tissues, which indicates that the MBF can be used as an index for discriminating differences in the microcirculatory conditions between acupoints and their surrounding tissues [26]. It has also been shown that acupuncture can not only increase general circulation [27] and circulation in specific organs [28] but also change the skin microcirculation as well [19, 24, 29, 30]. When an acupoint was stimulated adequately, the blood perfusion of this point continued to increase whereas the blood perfusion of nonacupoint only changed slightly by the same acupuncture stimulation [31]. These results indicated that the blood perfusion in acupoints can be recommended as candidate for acupuncture effect evaluation.

Our previous study has shown that ipsilateral acupoint stimulation could result in an increase of blood perfusion in contralateral side. But the lateralized characteristic is still not clear. This study indicated that the stimulation effect was different in different intervention groups. After stimulation of right LI4, the amplification effect on blood perfusion in contralateral is better than that in other two groups. It means under resting condition, the mean blood flux in both Hegu acupoints is symmetrical; after stimulating either side Hegu acupoint, this symmetry is broken. As a result, the MBF in the contralateral acupoint was amplified. But this amplification effect is different in different groups, which might be another phenomenon of symmetry breaking on a high level.

According to our previous study [32], under anesthesia condition, thermostimulation has no effect on the blood perfusion in the contralateral side foot. These results indicated that this asymmetry phenomenon was strengthened by the anesthesia. In other words, synchronous changes of bilateral blood perfusion might be related to the wakefulness status. Although it is difficult to explore the reasons, we think it might be related to the asymmetry of brain.

## Figures and Tables

**Table 1:** Subjects' demographic data.

Group	*n*	Gender (female/male)	Age (years, mean ± SD)
AL group	40	34/6	25.34 ± 1.77
AR group	40	25/15	25.85 ± 1.24
Control group	40	34/6	25.33 ± 1.69

AL: acupuncture left Hegu point; AR: acupuncture right Hegu point.

**Table 2:** Mapping value distribution center and signal-noise ratio in different groups.

Original input/output	Intervention method	Distribution center (PU)
*f*_*AL*⁡_(*k*) → *f*_AR_(*k*)	Acup. Left	255.41
*f*_BL_(*k*) → *f*_BR_(*k*)	Acup. Right	591.01
*f*_CL_(*k*) → *f*_CR_(*k*)	No acup.	71.58
*f*_AR_(*k*) → *f*_*AL*⁡_(*k*)	Acup. Left	222.32
*f*_BR_(*k*) → *f*_BL_(*k*)	Acup. Right	965.81
*f*_CR_(*k*) → *f*_CL_(*k*)	No acup.	96.33
